# Homeostatic activity regulation as a mechanism underlying the effect of brain stimulation

**DOI:** 10.1186/s42234-019-0032-0

**Published:** 2019-09-25

**Authors:** Zhi Chai, Cungen Ma, Xiaoming Jin

**Affiliations:** 1 Neurobiology Research Center, College of Basic Medicine, Shanxi University of Chinese Medicine, Taiyuan, 030619 China; 2 Department of Anatomy, Cell Biology and Physiology, Department of Neurological Surgery, Spinal Cord and Brain Injury Research Group, Stark Neurosciences Research Institute, Indiana University School of Medicine, 320 West 15th Street, NB 500C, Indianapolis, IN 46202 USA

**Keywords:** Homeostatic synaptic plasticity, Hyperexcitability, Brain stimulation, Epilepsy, Neuropathic pain, Tinnitus, Traumatic brain injury, Stroke

## Abstract

Hyperexcitability of the neural network often occurs after brain injuries or degeneration and is a key pathophysiological feature in certain neurological diseases such as epilepsy, neuropathic pain, and tinnitus. Although the standard approach of pharmacological treatments is to directly suppress the hyperexcitability through reducing excitation or enhancing inhibition, different techniques for stimulating brain activity are often used to treat refractory neurological conditions. However, it is unclear why stimulating brain activity would be effective for controlling hyperexcitability. Recent studies suggest that the pathogenesis in these disorders exhibits a transition from an initial activity loss after acute injury or progressive neurodegeneration to subsequent development of hyperexcitability. This process mimics homeostatic activity regulation and may contribute to developing network hyperexcitability that underlies neurological symptoms. This hypothesis also predicts that stimulating brain activity should be effective in reducing hyperexcitability due to homeostatic activity regulation and in relieving symptoms. Here we review current evidence of homeostatic plasticity in the development of hyperexcitability in some neurological diseases and the effects of brain stimulation. The homeostatic plasticity hypothesis may provide new insights into the pathophysiology of neurological diseases and may guide the use of brain stimulation techniques for treating them.

## Introduction

Hyperexcitability and excessive abnormal activity of the neural network is a common pathophysiological mechanism underlying many neurological disorders such as epilepsy, neuropathic pain, tinnitus, and Alzheimer’s disease (Latremoliere and Woolf 2009; Eggermont 2005; Badawy et al. 2009a; Vossel et al. 2017). The observation of such hyperexcitability naturally leads to a treatment strategy that targets to directly inhibiting neuronal activity so that a normal level of activity and neurological functions will be recovered and maintained. In fact, modern pharmaceutical industry is dominated by efforts to develop drugs to inhibit different components of neural circuits. Many drugs work by inhibiting neuronal activity and excitability. However, this strategy does not always work successfully. Because many patients with neurological disorders are refractory to conventional drug treatments, more radical and invasive therapeutic approaches are often required for symptom control. For example, about one third of patients with neuropathic pain or acquired epilepsy cannot be effectively controlled with the best of existing drug treatments (Finnerup et al. 2015; Yoo and Panov 2019).

Paradoxically, brain stimulation that enhances neuronal activity is also found to be effective for treating these hyperexcitable neurological diseases (De Ridder et al. 2007; Treister et al. 2013; Morrell 2011). How can either inhibiting or stimulating neuronal activity be effective in controlling these diseases? While various hypotheses have been proposed to explain the therapeutic mechanism, the direct effect of brain stimulation on the stimulated network is not well understood. A deep understanding of causes that lead to hyperexcitability may provide novel insight on the mechanism and treatment strategy. Because these neurological disorders start with an acute or chronic injury, progressive degeneration of neurons, or loss of afferent input, a homeostatic plasticity mechanism may play a role in the development and maintenance of brain hyperexcitability. This hypothesis also supports that stimulating brain activity can control aberrant hyperexcitability through compensating the lost activity to reduce homeostatic hyperexcitability. Recent studies on diseases such as neuropathic pain, acquired epilepsy, and tinnitus support a role of homeostatic plasticity mechanism and demonstrate the effectiveness of targeting excitatory activity for disease treatment. Here we review observations on neurological diseases that feature circuit hyperexcitability and the effectiveness of brain stimulation on them. We summarize recent progress on using homeostatic plasticity to explain the mechanism and to guide developing novel treatment strategies in the future.

## Hyperexcitability is a key neurophysiological change in some neurological diseases

Network hyperexcitability occurs in many neurological disorders such as epilepsy, traumatic brain injury (TBI), chronic pain, migraine, stroke, tinnitus, and Alzheimer’s disease (AD). These conditions involve injuries or pathologies of either the central nervous system (CNS) (e.g. epilepsy) or peripheral nervous system (PNS) (e.g. neuropathic pain). In terms of etiology, there are acute injuries such as TBI, spinal cord injury, and stroke, or chronic damage or neurodegeneration such as AD.

**Fig. 1:**
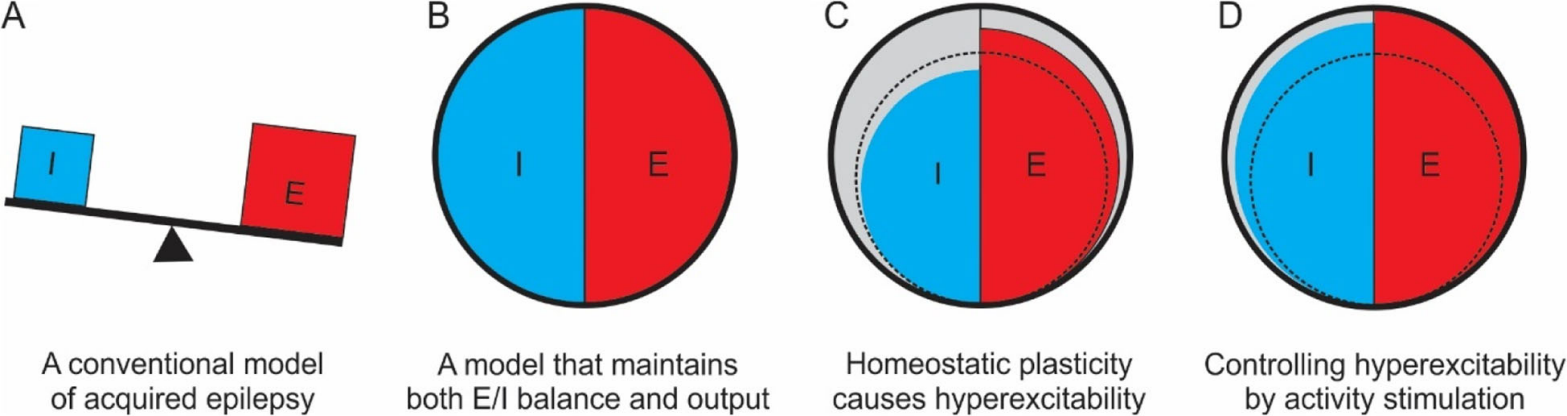
A schematic presentation of the homeostatic plasticity mechanism. **a**. Reduced inhibition (I) and/or enhanced excitation (E) that leads to hyperexcitability is a widely accepted mechanism of acquired epilepsy. **b**. We propose that the brain needs to not only maintain a balance between E and I, but also maintain a set level of functional output (i.e. neuronal activity, represented here as filling the circle). **c**. Injuries and other brain pathologies cause loss of neurons and neuronal activity (dotted circle). To compensate for the lost function, the neural network would use the homeostatic plasticity mechanism to scale up excitation (E) (and may simultaneously reduce inhibition (I)) to attempt to reach a set level of neuronal activity, which causes hyperexcitability simultaneously. **d.** Controlling hyperexcitability may be achieved by stimulating neuronal activity after injuries or degeneration, which will promote functional recovery and suppress intrinsic homeostatic activity regulation, and increase activity-dependent enhancement of inhibition

Epilepsy has hyperexcitability as its defining pathophysiological feature, with epileptic seizures resulting from an imbalance between excitation and inhibition. Hyperexcitability has been observed in many different types of epilepsies (Badawy et al. 2009a; Bauer et al. 2014). Enhanced excitation or reduced inhibition is generally regarded as an important basic mechanism in the generation of epileptic seizures (Badawy et al. 2009b; Scharfman 2007) (Fig. 1a). Moreover, most antiepileptic drugs work by enhancing inhibition and/or reducing excitation (Stafstrom 2010; White et al. 2007).

Recovery following stroke or TBI involves development of hyperexcitability. In the peri-infarct region of stroke, transient appearance of low-frequency spontaneous activity (0.1–1.0 Hz) occurs earlier after injury, followed by development of hyperexcitability in the following weeks (Carmichael and Chesselet 2002). High discharge frequency in the perilesional region peaks in 3–7 days post-stroke and maintains higher for up to 4 months (Neumann-Haefelin et al. 1995; Schiene et al. 1996). In a mouse model of permanent ligation of the middle cerebral artery, hyperexcitability of sensorimotor cortex also develops on the intact contralateral cortex in 2–6 weeks after stroke (Barios et al. 2016). Similarly, TBI is known to cause hyperexcitability of neocortex and hippocampus by increasing glutamate signaling, enhancing synaptic bursting, impairing GABAergic inhibition, and inducing epileptiform activity (Nichols et al. 2015; Cantu et al. 2015; Golarai et al. 2001; Hoffman et al. 1994).

Neuropathic pain originates from a primary lesion of the somatosensory nervous system such as nerve or spinal cord injury, which causes peripheral and central sensitization of the nociceptive pathways (Latremoliere and Woolf 2009). The resulting neuronal hyperexcitability and ectopic spontaneous firing of the nociceptive pathways are believed to be its key underlying neurophysiological mechanism. In tibial nerve injury model of neuropathic pain, optical imaging of voltage sensitive dye revealed increased optical intensity and an enlarged area of activation in the primary somatosensory cortex (S1) of neuropathic rats during electrical stimulation (Cha et al. 2009; Xiong et al. 2017). Hyperexcitability is also observed in anterior cingulate cortex through regulating intrinsic neuronal excitability and synaptic transmission in models of neuropathic pain (Blom et al. 2014; Gao et al. 2016; Yang et al. 2018).

Tinnitus is the perception of a sound in the absence of acoustic stimulation. Cochlear damage and hearing loss can lead to tinnitus and abnormally increased spontaneous firing rates, synchronization of neurons, and elevated AMPA receptor mRNA expression and reduced GABAA receptor mRNA expression in the auditory pathway, including the primary auditory and associated cortices (Bartels et al. 2007; Elgoyhen et al. 2015; Balaram et al. 2019). Interestingly, in a blast brain injury model in rats, spontaneous activity in auditory cortex in the tinnitus-positive rats show robust hyperactivity at all frequency regions in 3 months after injury (Luo et al. 2017). In a hearing loss model, neurons in the auditory cortex that represent the hearing-loss frequencies have reduced inhibitory synaptic transmission, unaltered excitatory synaptic transmission, and behavioral signs of tinnitus with the pitch in the hearing-loss frequency range (Yang et al. 2011).

Alzheimer’s disease (AD) is a neurodegenerative disorder characterized by dementia and progressive memory loss. Network hyperexcitability and epilepsy is a feature of AD in patients as well as in numerous mouse models (Palop et al. 2007; Kazim et al. 2017; Garcia-Cabrero et al. 2013). Interictal spikes are seen in a high percentage of AD patients who have no prior history of clinical seizures (Vossel et al. 2016). Nonictal network hyperactivity has been detected with fMRI in individuals at risk of developing dementia, such as in those carrying the APOE4 allele (Filippini et al. 2009) and in patients with mild cognitive impairment (Dickerson et al. 2005).

Additionally, other neurological disorders feature development of network hyperexcitability such as migraine and autism spectrum disorders including fragile X syndrome (Burstein et al. 2015; Zarcone and Corbetta 2017; Takarae and Sweeney 2017).

The hyperexcitability in these neurological conditions can be induced by various neurophysiological alterations, which include sprouting of excitatory axons, changes in intrinsic excitability of pyramidal neurons, insertion of AMPA receptors and enhanced excitatory synaptic transmission, reduced number of interneurons, impaired inhibitory synapses, impaired efficacy of inhibition due to chloride potassium transporter (Becker 2018; Prince et al. 2009). Although these mechanisms explain hyperexcitability at certain specific time points after a latent period following acute injury (e.g. posttraumatic epilepsy and stroke) or during the chronic phase of progressive neurodegeneration (e.g. AD), they do not elucidate why a damaged brain tends to become hyperexcitable. In this regard, a homeostatic plasticity mechanism may provide a useful model to explain the development of network hyperexcitability.

## Homeostatic plasticity drives the development of network hyperexcitability

Homeostatic plasticity is the intrinsic capability of the neural network to maintain a relatively constant level of activity in response to an imposed increase or decrease in neuronal activity (Turrigiano et al. 1998). For example, when a cortical network loses activity or afferent input, it responds with enhanced excitatory synaptic strength and intrinsic excitability and/or a reduction in synaptic inhibition to maintain a relatively constant level of activity (Turrigiano et al. 1998; Davis and Bezprozvanny 2001). Although homeostatic plasticity has been extensively studied in cultured neurons, brain slices, and more recently in visual cortex in vivo *(*Keck et al. 2013*;* Hengen et al. 2013*;* Goel and Lee 2007*;* Echegoyen et al. 2007*)*, its role in neurological disorders only begins to be revealed. Based on the homeostatic plasticity mechanism, we hypothesize that neural network must not only keep a dynamic balance between excitation and inhibition, but also maintain a certain level of activity as its functional output (Fig. 1b). Such homeostatic regulation may serve as a compensatory mechanism after brain injuries or neurodegeneration. Because hyperexcitability often develops from an acute (e.g. TBI and neuropathic pain) or chronic (e.g. AD) loss of neurons and synapses, abnormal homeostatic plasticity in response to the lesions and activity loss likely contributes to the development of hyperexcitability that underlies the symptoms (Fig. 1c). Indeed, sensory deprivation due to PNS injury is identical to some classical animal models for inducing homeostatic plasticity.

Homeostatic synaptic plasticity may be a driving force that underlies the development of acquired epilepsy, which usually develops following an initial insult such as TBI or status epilepticus (Houweling et al. 2005; Avramescu and Timofeev 2008; Dinocourt et al. 2011). Brain injury, particularly severe TBI and penetrating TBI, causes neuronal death, tissue damage, and an initial loss of activity in surviving neurons (Ping and Jin 2016; Alves et al. 2005). Lower action potential firing rates are recorded in the lateral fluid percussion and undercut models of TBI in vivo (Alves et al. 2005; Timofeev et al. 2000). Pharmacological blockade of neuronal activity of hippocampal neurons in vitro or in vivo for a few days leads to hyperexcitability with increased glutamatergic transmission, decreased GABAergic synaptic inputs, and epileptogenesis (Trasande and Ramirez 2007). In the hippocampus of developing animals, chronic blockade of activity with tetrodotoxin or a lesion produces chronic focal seizures accompanied by axon sprouting and increased intrinsic excitability (McKinney et al. 1997; Bausch et al. 2006). Similarly, neuronal activity is also depressed following brain ischemia (Heiss et al. 1976), which is followed by development of hyperexcitability.

Deprivation of peripheral input by activity blockade, amputation, or nervous lesion may cause homeostatic hyperexcitability of cortical network in developing or adult brain (Xiong et al. 2017; Wang and Thompson 2008). Such homeostatic plasticity regulation may underlie the development of hyperexcitability in neuropathic pain. During the earlier time period after spinal cord injury, slower and more silent overall cortical spontaneous activity is recorded in the deafferented cortex as well as in the neighboring cortex, representing a switch to a slow-wave network activity (Boord et al. 2008). In a spinal cord ischemia model of neuropathic pain, in vivo two-photon imaging demonstrated that initial activity loss occurs in 6 h after injury in cortical layer II/III pyramidal neurons of the primary somatosensory cortex, followed by recovery and hyperactivity in 48 h (Xiong et al. 2017). Because development of neuropathic pain reflects a transition from an initial loss of neuronal activity due to a primary lesion (e.g. nerve or spinal cord injury (SCI)) to a state of hyperexcitability of the affected neural network, this process is identical to the classical model of homeostatic plasticity.

Homeostatic plasticity is also suggested to contribute to hyperexcitability in auditory pathway in tinnitus (Yang et al. 2011) {Auerbach, 2014 #38}. A computational study suggested that homeostatic compensation leads to hyperactivity of the model neurons when a normal ratio between mean and spontaneous firing rate of the auditory nerve is decreased due to a loss of outer hair cells or damage to hair cell stereocilia. Homeostasis can also amplify non-auditory inputs, which then contribute to hyperactivity (Schaette and Kempter 2006).

## Homeostatic plasticity supports the effectiveness of brain stimulation

The idea that pathological hyperexcitability originates from homeostatic plasticity suggests that it is not sufficient to directly suppress hyperexcitability by blocking excitation or enhancing inhibition. If a loss of neuronal activity caused by CNS or PNS injuries induces homeostatic hyperexcitability and neurological disorders, then stimulating activity at an earlier time period following injury should suppress pathological homeostatic regulation/compensation and prevent the development of neurological diseases (e.g. acquired epilepsy after brain injury) (Fig. 1d). Furthermore, because the primary lesion or pathology in the etiology of these neurological disorders is often permanent or progressive, such homeostatic compensation is likely a constant or progressive process so that the deafferented or injured brain circuits can maintain a set level of activity. In that case, stimulating brain activity will relieve the constant burden of homeostatic regulation so that hyperexcitability is reduced and activity of neural circuits is reversed to a relatively normal activity state (Fig. 1d). Therefore, stimulating activity should be also effective in controlling or reversing neurological conditions that have already developed through homeostatic regulation. Below we summarize current evidence that supports this idea (Table 1).

**Table 1:** Effects of activity enhancement on neurological disorders featuring hyperexcitability

Condition	Model or patients	Treatment	Effect	References
Acquired epilepsy	Multi-electrode arrays recording of neuronal culture in vitro.	Electrical stimulation (0.05–50 Hz)	Higher stimulation frequency transforms burst activity to dispersed spiking reminiscent of the awake cortex in vivo	(Madhavan et al. 2006; Wagenaar et al. 2005)
	Temporal lobe epilepsy in rats in vivo	Electrical stimulation of subiculum after kindling or pilocarpine injection	1 Hz stimulation retarded progression of kindling seizures and inhibited chronic spontaneous pilocarpine-induced seizures.	(Han et al. 2014; Zhong et al. 2012)
	191 patients with refractory partial-onset seizures. A double-blind, randomized, controlled trial	Open-loop responsive cortical stimulation for 1 month	Reduction in seizure frequency in the treatment group (− 37.9%) than control group (− 17.3%).	(Morrell 2011)
	Alpha (2)-adrenoceptor antagonist atipamezole	Treatment started 1 week after SE induction and lasted for 9 weeks.	Lower seizure frequency and severity, and milder cell damage and mossy fiber sprouting in treatment group.	(Pitkanen et al. 2004)
Neuropathic pain	Spared tibial nerve injury and transient spinal cord ischemia models of neuropathic pain in mice	S1 optogenetic stimulation for 1 week, or S1 activity enhancement by bicuculline.	Reduced pain-like behavior in both models and reduced S1 neuronal [80]excitability.	(Xiong et al. 2017).
	Eight intractable neuropathic pain patients	TMS (1–50 Hz for 1 h) or electrical stimulation (4–8 Hz) for 1 month.	Significant pain relief in all patients.	(De Ridder et al. 2007)
Tinnitus	43 intractable tinnitus patients	Implanted electrodes in the primary auditory cortex or secondary auditory cortex	67% of patients improved with average tinnitus reduction of 53%. Burst stimulation has better effect than tonic stimulation.	(De Ridder et al. 2011) (Meng et al. 2011).
	163 tinnitus patients	rTMS at 1 Hz (2000 stimuli, 110% motor threshold) or sham stimulation	This protocol has no effect.	(Landgrebe et al. 2017)
	Ten tinnitus patients	rTMS at 1 Hz on auditory cortex for 5 consecutive days	Improvement was associated with increases intracortical inhibition, intracortical facilitation, and prolongation of cortical silent period.	(Langguth et al. 2007)
	Tinnitus induced by tone exposure in rats	Auditory cortex electrical stimulation with electrical array.	Tinnitus is suppressed and hearing is improved at the central level	(Zhang et al. 2011)

The predicted effectiveness of activity enhancement on disease prevention and treatment is supported by some evidence from previous clinical and animal studies. Electrical stimulation is effective in reducing bursting activity in neuronal culture in vitro (Madhavan et al. 2006; Wagenaar et al. 2005) and in enhancing neuronal plasticity and synaptic reorganization and controlling partial seizures in drug resistant patients in vivo (Ziemann et al. 2002; Demirtas-Tatlidede et al. 2011). Electrical stimulation of hippocampus has also been demonstrated to be effective and safe for controlling refractory temporal lobe epilepsy (Han et al. 2014). A recent double-blind, randomized, controlled trial in patients with refractory partial-onset seizures suggested that open loop cortical stimulation for 1 month resulted in a significant reduction in mean seizure frequency in the treatment group compared with that in the sham group (37.9% versus 17.3%) (Morrell 2011). However, evidence that specifically supports a role of homeostatic plasticity in preventing acquired epileptogenesis or controlling epileptic seizures is still not available.

Activity enhancement can also be achieved by pharmacologically modulating excitatory or inhibitory components of neural network. Treatment of cultured hippocampal slices with bicuculline for 1 week greatly diminishes the intensity of epileptiform activity that could be induced (Swann et al. 2007). For acquired epilepsy, cannabinoid antagonist SR141716A and alpha (2)-adrenoceptor antagonist atipamezole are both proconvulsant, but their application immediately after brain insults prevents the development of hyperexcitability or reduces seizure frequency and severity in animal models of epilepsy (Echegoyen et al. 2009; Pitkanen et al. 2004).

Auditory cortical stimulation may be a valuable treatment option for severe intractable tinnitus. In severe cases of intractable tinnitus, 37% of patients were responsive to tonic auditory cortex stimulation via implanted electrodes in the primary auditory cortex or overlying the secondary auditory cortex. A half of the 63% non-responders became responsive after switching to burst stimulation (De Ridder et al. 2011). Burst stimulation is capable of suppressing tinnitus in more patients more effectively than tonic stimulation, especially for noise-like tinnitus (Meng et al. 2011). However, non-invasive brain stimulation using repetitive transcranial magnetic stimulation (rTMS) have shown mixed results on tinnitus, with some studies showing significant improvement in the severity of tinnitus while the others having no significant effect (Meng et al. 2011; Londero et al. 2018; Landgrebe et al. 2017). Since different stimulation parameters and study designs affect the efficacy of rTMS and treatment outcome, further basic and translational studies are needed to elucidate the efficacy and mechanism of rTMS for tinnitus.

Enhancing brain activity after peripheral lesion can control cortical hyperexcitability and reduce pain. We recently showed that using optogenetic stimulation or a GABA_A_ receptor antagonist to enhance cortical layer V pyramidal neuron activity in S1 resulted in reduced pain-like behavior in a transient spinal cord ischemia model and a tibial nerve injury model of neuropathic pain. The stimulation directly reduced hyperexcitability of the S1 through decreasing excitatory synaptic transmission and increasing the threshold of action potential firing of the related cortical neurons (Xiong et al. 2017). Clinical studies demonstrated that stimulating motor cortex or S1 using rTMS (5–20 Hz) is effective in controlling refractory chronic pain including neuropathic pain and phantom pain (De Ridder et al. 2007; Lima and Fregni 2008). Although the underlying mechanisms of brain stimulation on refractory pain are unclear and may involve inhibiting the nociceptive pathway (e.g. thalamus) and activating descending pain modulation (Treister et al. 2013), homeostatic plasticity regulation may provide a novel explanation for the effect.

## Perspectives and conclusions

Research on homeostatic plasticity has been mainly focused on its basic physiological role and mechanisms, while sensory deprivation is often used as a tool for inducing homeostatic plasticity. Recent studies have extended to various neurological conditions such as neuropathic pain and acquired epilepsy. Because neuronal death and degeneration is a common etiology of many neurological disorders and the CNS cannot compensate for the lost function through regeneration, homeostatic plasticity likely plays a critical role in functional compensation which often leads to pathological hyperexcitability. Future study to understand its role and mechanism in different neurological diseases is important for their prevention and treatment, which is particularly true since a high percentage of neurological patients are refractory to current drug treatment.

Establishing the role of homeostatic plasticity in the etiology of neurological diseases has broad and important significance. This mechanism supports that stimulating excitatory activity will be effective in the prevention and treatment of these diseases, which will open a new direction for future research. In vitro and in vivo electrophysiological recoding and recent activity imaging techniques, such as calcium imaging in GCaMP6 expressing neurons, will allow us to characterize potential homeostatic activity regulation in different models of neurological diseases. Optogenetic stimulation and chemogenetic stimulation such as designer receptor exclusively activated by designer drugs (DREADD) provide powerful tools to specifically activate excitatory or inhibitory neurons for testing the homeostatic plasticity hypothesis. While much is understood about the molecular mechanisms of homeostatic plasticity {Li, 2019 #37}, whether these mechanisms are involved in related neurological conditions needs to be further studied. The homeostatic plasticity hypothesis may guide developing effective brain stimulation protocols for disease treatment or prevention. For example, we found that early optogenetic stimulation of cortical excitatory neurons after brain injury is effective in preventing posttraumatic epileptogenesis (unpublished data). Because the mechanism of the brain stimulation is not clear, there is no theory to guide the development of brain stimulation protocols including the frequency, duration, and target. Treatment based on homeostatic plasticity would require that the frequency and pattern of cortical stimulation be similar to physiological activity and longer duration of stimulation may be more beneficial. It will be important to determine whether using brain stimulating protocols based on homeostatic plasticity hypothesis will be more effective than ones based on experience. The hypothesis also supports that combining cortex stimulation with rehabilitation or peripheral stimulation (Levy et al. 2016) may have good effects by compensating the lost activity and reducing hyperexcitability. For example, pairing electrical stimuli and external stimuli (noise) in tinnitus patients is shown to drive cortical activity more efficiently and improve the outcome (De Ridder et al. 2014). In addition to various brain stimulation techniques, drugs that stimulate the injured neuronal circuit should also be effective in controlling neurological disorders including epilepsy and neuropathic pain. Obviously, research in this direction has important translational significance. Particularly, when a neurological disorder is refractory to conventional treatments that target to directly inhibit hyperexcitability fail, a strategy that aims to enhance excitatory activity for symptom control may be effective. Future study may focus on determining whether activating AMPA or NMDA receptors, or inhibiting GABAergic inhibition will be effective in controlling hyperexcitability and relieving related neurological symptoms. Achieving a balance between enhancing activity and avoiding excitotoxicity and seizures will be a key consideration in this line of study.

In conclusion, homeostatic plasticity regulation can explain why activity enhancement through various techniques of brain stimulation is effective for treating hyperexcitable neurological diseases. The homeostatic plasticity mechanism may also provide guidance on designing protocols for brain stimulation. Such stimulation will enhance and normalize spontaneous activity and improve functional connectivity of the related network, leading to symptom relief and functional improvement. Because the activity stimulation strategy is consistent with the intrinsic need of the body to compensate for lost function, it may be more effective and longer lasting in controlling hyperexcitable neurological disorders, including the refractory ones.

## Data Availability

N/A

## Abbreviations

AD: Alzheimer’s disease
CNS: Central nervous system
PNS: Peripheral nervous system
rTMS: Repetitive transcranial magnetic stimulation (rTMS)
S1: Primary somatosensory cortex
SCI: Spinal cord injury
TBI: Traumatic brain injury

## References

[CR1] Alves OL, Bullock R, Clausen T, Reinert M, Reeves TM (2005). Concurrent monitoring of cerebral electrophysiology and metabolism after traumatic brain injury: an experimental and clinical study. J Neurotrauma.

[CR2] Avramescu S, Timofeev I (2008). Synaptic strength modulation after cortical trauma: a role in epileptogenesis. J Neurosci.

[CR3] Auerbach BD, Rodrigues PV, Salvi RJ. Central gain control in tinnitus and hyperacusis. Front Neurol. 2014;5:206. doi: 10.3389/fneur.2014.00206.PubMed PMID: 25386157; PubMed Central PMCID: PMCPMC4208401. 10.3389/fneur.2014.00206 PMC4208401 25386157

[CR4] Badawy RA, Harvey AS, Macdonell RA (2009). Cortical hyperexcitability and epileptogenesis: understanding the mechanisms of epilepsy - part 1. J Clin Neurosci.

[CR5] Badawy RA, Harvey AS, Macdonell RA (2009). Cortical hyperexcitability and epileptogenesis: understanding the mechanisms of epilepsy - part 2. J Clin Neurosci.

[CR6] Balaram P, Hackett TA, Polley DB (2019). Synergistic Transcriptional Changes in AMPA and GABAA Receptor Genes Support Compensatory Plasticity Following Unilateral Hearing Loss. Neuroscience.

[CR7] Barios JA, Pisarchyk L, Fernandez-Garcia L, Barrio LC, Ramos M, Martinez-Murillo R (2016). Long-term dynamics of somatosensory activity in a stroke model of distal middle cerebral artery oclussion. J Cereb Blood Flow Metab.

[CR8] Bartels H, Staal MJ, Albers FW (2007). Tinnitus and neural plasticity of the brain. Otol Neurotol.

[CR9] Bauer PR, Kalitzin S, Zijlmans M, Sander JW, Visser GH (2014). Cortical excitability as a potential clinical marker of epilepsy: a review of the clinical application of transcranial magnetic stimulation. Int J Neural Syst.

[CR10] Bausch SB, He S, Petrova Y, Wang XM, McNamara JO (2006). Plasticity of both excitatory and inhibitory synapses is associated with seizures induced by removal of chronic blockade of activity in cultured hippocampus. J Neurophysiol.

[CR11] Becker AJ (2018). Review: animal models of acquired epilepsy: insights into mechanisms of human epileptogenesis. Neuropathol Appl Neurobiol.

[CR12] Blom SM, Pfister JP, Santello M, Senn W, Nevian T (2014). Nerve injury-induced neuropathic pain causes disinhibition of the anterior cingulate cortex. J Neurosci.

[CR13] Boord P, Siddall PJ, Tran Y, Herbert D, Middleton J, Craig A (2008). Electroencephalographic slowing and reduced reactivity in neuropathic pain following spinal cord injury. Spinal Cord.

[CR14] Burstein R, Noseda R, Borsook D (2015). Migraine: multiple processes, complex pathophysiology. J Neurosci.

[CR15] Cantu D, Walker K, Andresen L, Taylor-Weiner A, Hampton D, Tesco G (2015). Traumatic Brain Injury Increases Cortical Glutamate Network Activity by Compromising GABAergic Control. Cereb Cortex.

[CR16] Carmichael ST, Chesselet MF (2002). Synchronous neuronal activity is a signal for axonal sprouting after cortical lesions in the adult. J Neurosci.

[CR17] Cha MH, Kim DS, Cho ZH, Sohn JH, Chung MA, Lee HJ (2009). Modification of cortical excitability in neuropathic rats: a voltage-sensitive dye study. Neurosci Lett.

[CR18] Davis GW, Bezprozvanny I (2001). Maintaining the stability of neural function: a homeostatic hypothesis. Annu Rev Physiol.

[CR19] De Ridder D, De Mulder G, Menovsky T, Sunaert S, Kovacs S (2007). Electrical stimulation of auditory and somatosensory cortices for treatment of tinnitus and pain. Prog Brain Res.

[CR20] De Ridder D, Vanneste S, Engineer ND, Kilgard MP (2014). Safety and efficacy of vagus nerve stimulation paired with tones for the treatment of tinnitus: a case series. Neuromodulation..

[CR21] De Ridder D, Vanneste S, Kovacs S, Sunaert S, Menovsky T, van de Heyning P (2011). Transcranial magnetic stimulation and extradural electrodes implanted on secondary auditory cortex for tinnitus suppression. J Neurosurg.

[CR22] Demirtas-Tatlidede Asli, Vahabzadeh-Hagh Andrew M., Bernabeu Montserrat, Tormos Jose M., Pascual-Leone Alvaro (2012). Noninvasive Brain Stimulation in Traumatic Brain Injury. Journal of Head Trauma Rehabilitation.

[CR23] Dickerson BC, Salat DH, Greve DN, Chua EF, Rand-Giovannetti E, Rentz DM (2005). Increased hippocampal activation in mild cognitive impairment compared to normal aging and AD. Neurology.

[CR24] Dinocourt Céline, Aungst Stephanie, Yang Kun, Thompson Scott M. (2011). Homeostatic increase in excitability in area CA1 after Schaffer collateral transection in vivo. Epilepsia.

[CR25] Echegoyen J, Armstrong C, Morgan RJ, Soltesz I (2009). Single application of a CB1 receptor antagonist rapidly following head injury prevents long-term hyperexcitability in a rat model. Epilepsy Res.

[CR26] Echegoyen J, Neu A, Graber KD, Soltesz I (2007). Homeostatic plasticity studied using in vivo hippocampal activity-blockade: synaptic scaling, intrinsic plasticity and age-dependence. PLoS One.

[CR27] Eggermont JJ (2005). Tinnitus: neurobiological substrates. Drug Discov Today.

[CR28] Elgoyhen AB, Langguth B, De Ridder D, Vanneste S (2015). Tinnitus: perspectives from human neuroimaging. Nat Rev Neurosci.

[CR29] Filippini N, MacIntosh BJ, Hough MG, Goodwin GM, Frisoni GB, Smith SM (2009). Distinct patterns of brain activity in young carriers of the APOE-epsilon4 allele. Proc Natl Acad Sci U. S. A..

[CR30] Finnerup NB, Attal N, Haroutounian S, McNicol E, Baron R, Dworkin RH (2015). Pharmacotherapy for neuropathic pain in adults: a systematic review and meta-analysis. Lancet Neurol.

[CR31] Gao SH, Wen HZ, Shen LL, Zhao YD, Ruan HZ (2016). Activation of mGluR1 contributes to neuronal hyperexcitability in the rat anterior cingulate cortex via inhibition of HCN channels. Neuropharmacology..

[CR32] Garcia-Cabrero AM, Guerrero-Lopez R, Giraldez BG, Llorens-Martin M, Avila J, Serratosa JM (2013). Hyperexcitability and epileptic seizures in a model of frontotemporal dementia. Neurobiol Dis.

[CR33] Goel A, Lee HK (2007). Persistence of experience-induced homeostatic synaptic plasticity through adulthood in superficial layers of mouse visual cortex. J Neurosci.

[CR34] Golarai G, Greenwood AC, Feeney DM, Connor JA (2001). Physiological and structural evidence for hippocampal involvement in persistent seizure susceptibility after traumatic brain injury. J Neurosci.

[CR35] Han CL, Hu W, Stead M, Zhang T, Zhang JG, Worrell GA (2014). Electrical stimulation of hippocampus for the treatment of refractory temporal lobe epilepsy. Brain Res Bull.

[CR36] Heiss WD, Hayakawa T, Waltz AG (1976). Cortical neuronal function during ischemia. Effects of occlusion of one middle cerebral artery on single-unit activity in cats. Arch Neurol.

[CR37] Hengen KB, Lambo ME, Van Hooser SD, Katz DB, Turrigiano GG (2013). Firing rate homeostasis in visual cortex of freely behaving rodents. Neuron..

[CR38] Hoffman SN, Salin PA, Prince DA (1994). Chronic neocortical epileptogenesis in vitro. J Neurophysiol.

[CR39] Houweling AR, Bazhenov M, Timofeev I, Steriade M, Sejnowski TJ (2005). Homeostatic synaptic plasticity can explain post-traumatic epileptogenesis in chronically isolated neocortex. Cereb Cortex.

[CR40] Kazim SF, Chuang SC, Zhao W, Wong RK, Bianchi R, Iqbal K (2017). Early-Onset Network Hyperexcitability in Presymptomatic Alzheimer's Disease Transgenic Mice Is Suppressed by Passive Immunization with Anti-Human APP/Abeta Antibody and by mGluR5 Blockade. Front Aging Neurosci.

[CR41] Keck T, Keller GB, Jacobsen RI, Eysel UT, Bonhoeffer T, Hubener M (2013). Synaptic scaling and homeostatic plasticity in the mouse visual cortex in vivo. Neuron..

[CR42] Landgrebe M, Hajak G, Wolf S, Padberg F, Klupp P, Fallgatter AJ (2017). 1-Hz rTMS in the treatment of tinnitus: a sham-controlled, randomized multicenter trial. Brain Stimul.

[CR43] Langguth B, Kleinjung T, Marienhagen J, Binder H, Sand PG, Hajak G (2007). Transcranial magnetic stimulation for the treatment of tinnitus: effects on cortical excitability. BMC Neurosci.

[CR44] Latremoliere A, Woolf CJ (2009). Central sensitization: a generator of pain hypersensitivity by central neural plasticity. J Pain.

[CR45] Levy RM, Harvey RL, Kissela BM, Winstein CJ, Lutsep HL, Parrish TB (2016). Epidural electrical stimulation for stroke rehabilitation: results of the prospective, multicenter, randomized, Single-Blinded Everest Trial. Neurorehabil Neural Repair.

[CR46] Li Jie, Park Esther, Zhong Lei R., Chen Lu (2019). Homeostatic synaptic plasticity as a metaplasticity mechanism — a molecular and cellular perspective. Current Opinion in Neurobiology.

[CR47] Lima MC, Fregni F (2008). Motor cortex stimulation for chronic pain: systematic review and meta-analysis of the literature. Neurology..

[CR48] Londero A, Bonfils P, Lefaucheur JP (2018). Transcranial magnetic stimulation and subjective tinnitus. A review of the literature, 2014-2016. Eur Ann Otorhinolaryngol Head Neck Dis.

[CR49] Luo H, Pace E, Zhang J (2017). Blast-induced tinnitus and hyperactivity in the auditory cortex of rats. Neuroscience..

[CR50] Madhavan R, Chao ZC, Wagenaar DA, Bakkum DJ, Potter SM (2006). Multi-site stimulation quiets network-wide spontaneous bursts and enhances functional plasticity in cultured cortical networks. Conf Proc IEEE Eng Med Biol Soc.

[CR51] McKinney RA, Debanne D, Gahwiler BH, Thompson SM (1997). Lesion-induced axonal sprouting and hyperexcitability in the hippocampus in vitro: implications for the genesis of posttraumatic epilepsy. Nat Med.

[CR52] Meng Z, Liu S, Zheng Y, Phillips JS (2011). Repetitive transcranial magnetic stimulation for tinnitus. Cochrane Database Syst Rev.

[CR53] Morrell MJ (2011). Responsive cortical stimulation for the treatment of medically intractable partial epilepsy. Neurology..

[CR54] Neumann-Haefelin T, Hagemann G, Witte OW (1995). Cellular correlates of neuronal hyperexcitability in the vicinity of photochemically induced cortical infarcts in rats in vitro. Neurosci Lett.

[CR55] Nichols J, Perez R, Wu C, Adelson PD, Anderson T (2015). Traumatic brain injury induces rapid enhancement of cortical excitability in juvenile rats. CNS Neurosci Ther.

[CR56] Palop JJ, Chin J, Roberson ED, Wang J, Thwin MT, Bien-Ly N (2007). Aberrant excitatory neuronal activity and compensatory remodeling of inhibitory hippocampal circuits in mouse models of Alzheimer's disease. Neuron..

[CR57] Ping X, Jin X (2016). Transition from initial Hypoactivity to hyperactivity in cortical layer V pyramidal neurons after traumatic brain injury in vivo. J Neurotrauma.

[CR58] Pitkanen A, Narkilahti S, Bezvenyuk Z, Haapalinna A, Nissinen J (2004). Atipamezole, an alpha (2)-adrenoceptor antagonist, has disease modifying effects on epileptogenesis in rats. Epilepsy Res.

[CR59] Prince DA, Parada I, Scalise K, Graber K, Jin X, Shen F (2009). Epilepsy following cortical injury: cellular and molecular mechanisms as targets for potential prophylaxis. Epilepsia.

[CR60] Schaette R, Kempter R (2006). Development of tinnitus-related neuronal hyperactivity through homeostatic plasticity after hearing loss: a computational model. Eur J Neurosci.

[CR61] Scharfman HE (2007). The neurobiology of epilepsy. Curr Neurol Neurosci Rep.

[CR62] Schiene K, Bruehl C, Zilles K, Qu M, Hagemann G, Kraemer M (1996). Neuronal hyperexcitability and reduction of GABAA-receptor expression in the surround of cerebral photothrombosis. J Cereb Blood Flow Metab.

[CR63] Stafstrom CE (2010). Mechanisms of action of antiepileptic drugs: the search for synergy. Curr Opin Neurol.

[CR64] Swann JW, Le JT, Lam TT, Owens J, Mayer AT (2007). The impact of chronic network hyperexcitability on developing glutamatergic synapses. Eur J Neurosci.

[CR65] Takarae Yukari, Sweeney John (2017). Neural Hyperexcitability in Autism Spectrum Disorders. Brain Sciences.

[CR66] Timofeev I, Grenier F, Bazhenov M, Sejnowski TJ, Steriade M (2000). Origin of slow cortical oscillations in deafferented cortical slabs. Cereb Cortex.

[CR67] Trasande CA, Ramirez JM (2007). Activity deprivation leads to seizures in hippocampal slice cultures: is epilepsy the consequence of homeostatic plasticity?. J Clin Neurophysiol.

[CR68] Treister R, Lang M, Klein MM, Oaklander AL (2013). Non-invasive transcranial magnetic stimulation (TMS) of the motor cortex for neuropathic pain-at the tipping point?. Rambam Maimonides Med J.

[CR69] Turrigiano GG, Leslie KR, Desai NS, Rutherford LC, Nelson SB (1998). Activity-dependent scaling of quantal amplitude in neocortical neurons. Nature..

[CR70] Vossel KA, Ranasinghe KG, Beagle AJ, Mizuiri D, Honma SM, Dowling AF (2016). Incidence and impact of subclinical epileptiform activity in Alzheimer's disease. Ann Neurol.

[CR71] Vossel KA, Tartaglia MC, Nygaard HB, Zeman AZ, Miller BL (2017). Epileptic activity in Alzheimer's disease: causes and clinical relevance. Lancet Neurol.

[CR72] Wagenaar DA, Madhavan R, Pine J, Potter SM (2005). Controlling bursting in cortical cultures with closed-loop multi-electrode stimulation. J Neurosci.

[CR73] Wang G, Thompson SM (2008). Maladaptive homeostatic plasticity in a rodent model of central pain syndrome: thalamic hyperexcitability after spinothalamic tract lesions. J Neurosci.

[CR74] White HS, Smith MD, Wilcox KS (2007). Mechanisms of action of antiepileptic drugs. Int Rev Neurobiol.

[CR75] Xiong W, Ping X, Ripsch MS, Chavez GSC, Hannon HE, Jiang K (2017). Enhancing excitatory activity of somatosensory cortex alleviates neuropathic pain through regulating homeostatic plasticity. Sci Rep.

[CR76] Yang S, Weiner BD, Zhang LS, Cho SJ, Bao S (2011). Homeostatic plasticity drives tinnitus perception in an animal model. Proc Natl Acad Sci U S A.

[CR77] Yang Z, Tan Q, Cheng D, Zhang L, Zhang J, Gu EW (2018). The Changes of Intrinsic Excitability of Pyramidal Neurons in Anterior Cingulate Cortex in Neuropathic Pain. Front Cell Neurosci.

[CR78] Yoo JY, Panov F (2019). Identification and treatment of drug-resistant epilepsy. Continuum (Minneap Minn).

[CR79] Zarcone D, Corbetta S (2017). Shared mechanisms of epilepsy, migraine and affective disorders. Neurol Sci.

[CR80] Zhang J, Zhang Y, Zhang X (2011). Auditory cortex electrical stimulation suppresses tinnitus in rats. J Assoc Res Otolaryngol.

[CR81] Zhong K, Wu DC, Jin MM, Xu ZH, Wang Y, Hou WW (2012). Wide therapeutic time-window of low-frequency stimulation at the subiculum for temporal lobe epilepsy treatment in rats. Neurobiol Dis.

[CR82] Ziemann U, Wittenberg GF, Cohen LG (2002). Stimulation-induced within-representation and across-representation plasticity in human motor cortex. J Neurosci.

